# How to Make Immunotherapy an Effective Therapeutic Choice for Uveal Melanoma

**DOI:** 10.3390/cancers13092043

**Published:** 2021-04-23

**Authors:** Mariarosaria Marseglia, Adriana Amaro, Nicola Solari, Rosaria Gangemi, Elena Croce, Enrica Teresa Tanda, Francesco Spagnolo, Gilberto Filaci, Ulrich Pfeffer, Michela Croce

**Affiliations:** 1 IRCCS Ospedale Policlinico San Martino, 16132 Genoa, Italy; MariaRosaria.Marseglia@hsanmartino.it (M.M.); adriana.amaro@hsanmartino.it (A.A.); nicola.solari@hsanmartino.it (N.S.); rosaria.gangemi@hsanmartino.it (R.G.); croceelena91@gmail.com (E.C.); enrica.tanda@gmail.com (E.T.T.); francesco.spagnolo85@gmail.com (F.S.); gfilaci@unige.it (G.F.); 2 Department of Internal Medicine and Medical Specialties (DiMI), University of Genoa, 16132 Genoa, Italy

**Keywords:** uveal, immunotherapy, *BAP1*, tumor microenvironment, anti-PD-1, anti-CTLA-4, TIL

## Abstract

**Simple Summary:**

Despite improvements in the early identification and successful control of primary uveal melanoma, 50% of patients will develop metastatic disease with only marginal improvements in survival. This review focuses on the tumor microenvironment and the cross-talk between tumor and immune cells in a tumor characterized by low mutational load, the induction of immune-suppressive cells, and the expression of alternative immune checkpoint molecules. The choice of combining different strategies of immunotherapy remains a feasible and promising option on selected patients.

**Abstract:**

Uveal melanoma (UM), though a rare form of melanoma, is the most common intraocular tumor in adults. Conventional therapies of primary tumors lead to an excellent local control, but 50% of patients develop metastases, in most cases with lethal outcome. Somatic driver mutations that act on the MAP-kinase pathway have been identified, yet targeted therapies show little efficacy in the clinics. No drugs are currently available for the *G protein alpha subunits* *GNAQ* and *GNA11*, which are the most frequent driver mutations in UM. Drugs targeting the YAP–TAZ pathway that is also activated in UM, the tumor-suppressor gene *BRCA1 Associated Protein 1* (*BAP1*) and the *Splicing Factor 3b Subunit 1* gene (*SF3B1*) whose mutations are associated with metastatic risk, have not been developed yet. Immunotherapy is highly effective in cutaneous melanoma but yields only poor results in the treatment of UM: anti-PD-1 and anti-CTLA-4 blocking antibodies did not meet the expectations except for isolated cases. Here, we discuss how the improved knowledge of the tumor microenvironment and of the cross-talk between tumor and immune cells could help to reshape anti-tumor immune responses to overcome the intrinsic resistance to immune checkpoint blockers of UM. We critically review the dogma of low mutational load, the induction of immune-suppressive cells, and the expression of alternative immune checkpoint molecules. We argue that immunotherapy might still be an option for the treatment of UM.

## 1. Introduction

Uveal melanoma (UM) is the most common intraocular malignancy of adulthood. UM originates from melanocytes of the uvea, including the iris, ciliary body, and retinal choroid. Despite improvements in early identification and successful control of the primary tumor, approximately 20–30% of the patients develop metastatic disease within 5 years from diagnosis, while at 15 years, the percentage rises to 45%. UM metastatic sites are the liver, lung, soft tissue, and bone [1,2]. Most frequently, metastases involve the liver as the first or only target tissue, and untreated patients have a mean survival time of about 2 months that rises to close to 6 months upon treatment [1,3,4]. Distinct UM subtypes with different clinical outcomes and prognoses have been defined on the basis of various pathological parameters, with the contribution of different genetic abnormalities, through studies of gene expression profiles and The Cancer Genome Atlas (TCGA). Several driver mutations have been found, involving mainly *G protein alpha subunits GNAQ* and *GNA11* or, in a minor fraction of UM cases, the *Cysteinyl Leukotriene Receptor 2* (*CYSLTR2*) [5], and the *Phospholipase C Beta 4* (*PLCB4*) [6] genes. Mutations in *GNAQ* and *GNA11* are present in 75–95% of cases and occur early in the development of UM [2,7]. These mutations are mutually exclusive and lead to the constitutive activation of G alpha protein, which in turn leads to the activation of several downstream effectors, thus promoting cell growth and proliferation [8]. GNAQ and GNA11 activate the Phospholipase C/Protein Kinase C (PLC/PKC) pathway and several downstream signaling pathways, including the Rapidly Accelerated Fibrosarcoma/mitogen-activated protein kinase kinase/extracellular signal-regulated kinase (RAF/MEK/ERK), Phosphoinositide 3-kinase/AKT Serine/Threonine Kinase/Mechanistic Target Of Rapamycin Kinase (PI3K/AKT/MTOR), and Trio Rho Guanine Nucleotide Exchange Factor/Ras homologue family member/Rac family small GTPase 1/Yes associated protein 1 (Trio/Rho/Rac/YAP1) pathways [2]. Several molecules, such as CXCR4, c-MET, Hypoxia Inducible Factor 1 (HIF-1), and insulin-like-growth factor-1 (IGF-1) are involved in UM metastatic progression and thus considered as a target for new treatments [2]. Additional mutations in the calcium-signaling pathway, to which also *GNAQ* and *GNA11* belong, might also influence tumorigenesis [9].

The monosomy of chromosome 3 [10,11], loss of chromosome 3 heterozygosity [12], and inactivating mutations of the *BRCA1-associated protein 1* (*BAP1*) oncosuppressor gene [13] are strongly associated with metastatic risk. On the contrary, somatic mutations in *Eukaryotic Translation Initiation Factor 1A X-Linked* (*EIF1AX*) and *Splicing Factor 3b subunit 1* (*SF3B1*) genes prevalently occur in UM with disomy 3 [14,15]. According to data from whole-genome sequencing (WGS) and Sanger sequencing, *SF3B1*, *EIF1AX*, and *BAP1* mutations classify UM patients in different categories with different survival and metastatic risk. *EIF1AX* mutations are not associated with risk of metastasis and show, similar to tumors without *BAP1* and *SF3B1* mutations, prolonged survival. UM-bearing mutated *SF3B1* undergoes metastatic progression later, and tumors with mutated *BAP1* metastasize early and rapidly progress with poor survival rates [16]. *BAP1* is a tumor-suppressor gene located on chromosome 3; it encodes a deubiquitinating enzyme with tumor-suppressive activity [17,18]. Inactivating mutations of *BAP1* occur in nearly half of UM patients and approximately 84% of metastatic cases [13]. *BAP1* loss-of-function mutations correlate with a distinct DNA methylation profile [19]. Finally, germline *BAP1* mutations are associated with an early and increased incidence of UM [20] but also with an increased incidence of other malignancies [21]. Many secondary mutations were found by next-generation sequencing to occur in UM patients in the same G-protein-related pathways known as drivers, in particular in the calcium-signaling pathway [9]. These secondary driver mutations are likely to affect tumor development and progression.

Amplifications of the long arm of chromosome 8 confer an increased risk of metastasis in UM. Several genes such as *V-Myc Avian Myelocytomatosis Viral Oncogene Homolog* (*MYC*) and *Ankyrin Repeat and PH Domain 1* (*ASAP1*), located on the long arm of chromosome 8 have been proposed as mediators of the effects of 8q amplification [22]. Chromosome 6p amplifications exert a protective effect yet the molecular basis thereof has not been fully elucidated [22].

It is widely accepted that tumor mutational burden is an important biomarker to predict response to immune checkpoint blockers (ICB) in tumors. In UM, both primary tumor and metastases carry one of the lowest mutation burdens in adult solid tumors [23]. UM displays a mean mutation rate of 0.5 mutations per megabase (Mb) [6], as opposed to 49.2 in cutaneous melanoma (CM) [24]. The role of UV light has been proposed as the major cause for the differences in UM and CM mutational burden and a UV-associated mutational signature is expressed in CM [9,24,25]. Metastases from iris-UM, though rare, display a higher mutation load than the average of UM [24,26], and they are also connected to a UV signature [24]. The presence of germline mutations of *methyl-CpG-binding domain protein 4* (*MBD4*) was detected in a group of UM patients who experienced a disease stabilization and prolonged survival after ICB immunotherapy [27,28], thus suggesting a role for *MBD4* as a new predictor of response to immunotherapy in UM [29]. *MBD4* is thought to act as a tumor suppressor gene; it is located on chromosome 3, and mutations have recently been identified in approximately 2% of UM characterized by a high mutational burden and hypermutated tumors [27,29].

Treatment of primary UM (P-UM) consists in surgery or radiation. It has a low local recurrence rate, but almost 50% of the patients develop metastatic disease, prevalently to the liver [1]. At present, there are no effective therapies for metastatic UM (M-UM), and most patients survive less than 12 months after diagnosis of metastases [30,31]. Different therapeutic strategies, including targeted, immunotherapeutic, chemotherapeutic, and epigenetic, have been or are currently being investigated. Among different strategies pursued in clinical trials for UM, immunotherapy was the most promising, given the striking impact it had on CM patients’ survival [32]. We refer to other recent reviews [33] for deeper insights into UM classification, epidemiology, genetic, and epigenetic [2,22,34], because this is beyond the purpose of this review.

In this paper, we review recent advances in innovative immune therapy options for UM in adjuvant and metastatic settings and develop perspectives for translating them in clinical practice. Special issues concerning an immune-suppressive tumor microenvironment (TME), poor mutational load and antigen expression, and signatures defining patients’ responses will be addressed to define new immune therapeutic strategies for M-UM.

## 2. Immunobiology of Uveal Melanoma

The Melanoma Antigen Gene (MAGE) family proteins, tyrosinase, and gp100 are UM tumor-associated antigens (TAA) that are recognized by cells of the immune system [35]. Indeed, peripheral CD8+ cells from UM patients and tumor-infiltrating lymphocytes (TILs) can lysate UM cells in vitro [36,37]. Nevertheless, the immune privilege of the eye allows UM cells to escape the control of the immune system.

The most frequent site of UM metastases is the liver, but the mechanism that guides the liver tropism of UM remains elusive. The immunomodulatory nature of the liver is determined by its exposure to food antigens, allergens, and low levels of endotoxins, deriving from the gut. The liver microenvironment is composed of resident non-immune and immune cells, such as hepatocytes, liver sinusoidal endothelial cells (LSECs), Kupffer cells (KCs), T, NK, and NKT cells that strictly regulate the balance between tolerance and the defense against pathogens. UM cells that have escaped from the eye find further protection in the immune-modulatory microenvironment of the liver. Detailed mechanisms of immunosuppression in the eye and the liver will be described below.

### 2.1. Immunosuppressive Mechanisms in the Eye

Different mechanisms may contribute to immune suppression in UM, among which the site in which UM arises. The eye is a physiologically immune-privileged organ in order to protect it from destructive inflammation that may impair vision. This immune-privilege is maintained through different mechanisms, among which physical barriers such as the blood–retina barrier and the absence of efferent lymphatics [38,39].

Anterior chamber-associated immune deviation (ACAID), though difficult to be studied in humans, has been shown in different animal models, and it is responsible for the induction of complex immunoregulatory mechanisms and cells [33,40]. Characteristic of ACAID are the inhibition of Th1 differentiation and delayed-type hypersensitivity (DTH) [41].

A general immunosuppressive milieu in the eye avoids non-specific inflammatory reactions and immune responses. It is caused by the release of soluble factors (i.e., transforming growth factor-beta, TGF-β [42]), low MHC expression, the presence of neuropeptides, and expression of FAS ligand [43]. Primed T cells, activated in vitro in the presence of the aqueous humor, were reprogrammed to TGF-β producing regulatory T cells (Treg) and acquired immunosuppressive skills [42]. The aqueous humor also contains the pleiotropic cytokine Macrophage Migration Inhibitory Factor (MIF), which promotes immune privilege by inhibiting NK cell activity [44]. Finally, iris and ciliary body epithelial cells can prevent T cell activation and proliferation via direct cell-to-cell contact [45]. Specifically, in P-UM, soluble HLA class I (sHLA-I) has been detected in the anterior chamber aqueous humor and has been considered a prognostically unfavorable sign that may influence local immune responses. Indeed, sHLA-I was detected in monosomy 3 tumors, with gain of 8q and loss of BAP1 protein expression known to have a poor prognosis [46]. The immune-suppressive microenvironment of the eye is assumed to generate a niche in which UM can grow and proliferate without the pressure of both innate and adaptive immune cells until it breaks the blood–retina barrier and disseminates. Innate cells, especially NK cells, are believed to be able to prevent metastases or to kill tumor cells in the blood before they could reach the liver [47]. However, after leaving the eye, the ability of UM cells to express pro-oncogenic molecules such as indoleamine dioxygenase-1 (IDO-1, [48]), MIF [49], and PD-L1 [50] enhance their metastatic potential.

### 2.2. Immunosuppressive Mechanisms in the Liver

Considering that metastatic disease in UM patients may be diagnosed many years after the primary tumor, it has been proposed that UM cells that leave the eye and reach the liver remain stable for years until proliferation occurs. This characteristic has been called “UM cell dormancy” and implies that the disease was already disseminated at the time of diagnosis [51]. Dormant UM cells are quiescent cells blocked in the cell cycle that only occasionally undergo cell division, which is an adaptive mechanism used by cells in a hostile microenvironment. Dormancy consists in the regulation of cellular proliferation and includes autophagy, interaction with the extracellular matrix, hypoxia, impaired angiogenesis, inflammation, and immunity [51,52]. Liver UM metastases have been described, based on their growth pattern, as either infiltrative or nodular. The infiltrative pattern is characterized by UM cells lacking vascular endothelial growth factor (VEGF) expression, invading liver sinusoidal space, and creating pseudo-sinusoidal spaces for oxygen and nutrient supply. Differently, the nodular growth pattern arises in the peri-portal area, involves portal veins and, as the lesion becomes hypoxic, cells express Matrix Metallopeptidase 9 (MMP9) and VEGF, thus developing angiogenetic properties [53].

UM cells become resistant to NK cell-mediated cytolysis in the metastatic niche in the liver by producing TGF-β upregulating MHC-I molecules [54] and downregulating NK activating ligands for NKG2D [55]. Hepatic stellate cells are supposed to contribute to UM niche in the liver; they are recruited by UM cells and secrete pro-inflammatory factors and collagen [56].

### 2.3. Tumor-Infiltrating Lymphocytes

The presence of TILs is a marker of good prognosis for many cancers but not in UM where it is associated with a poor prognosis [57,58]. Why this is so is not fully understood, as there are contradictory reports on the immune cell subtypes populating liver metastases in UM [59,60,61,62,63]. It is of note that most studies that tried to characterize the immunosuppressive environment in UM metastases have been performed at the transcriptomic level on only very few immune cells. Robertson et al. [19] proposed a stratification based on CD8+ T-cell immune infiltrates and an altered transcriptional immune profile for P-UM bearing monosomy 3 and *BAP1* loss of function mutations. Using RNA-seq analysis, they showed an upregulation of CD8+ T cell-related genes in almost 30% of monosomic UM that was not observed in disomic cases. In addition, genes involved in interferon-γ (IFN-γ) signaling, T cell invasion, cytotoxicity, and immunosuppression were co-expressed with *CD8A*, as well as with *HLA* genes [19].

Chromosome 8q amplification is related to macrophage infiltration, and the loss of *BAP1* expression is associated with T cell infiltration in UM [64]. TILs do not seem to be cytotoxic CD8+ but mostly regulatory CD8+ T lymphocytes [65]. Moreover, *BAP1* loss correlated with the upregulation of several genes associated with a suppressive immune response, including *HLA-DRA*, *CD38*, and *CD74*, both in primary and metastatic tumors. Digital spatial profiling, a genomic analysis that maintains the spatial information of UM metastases, showed tumor-associated macrophages (TAMs) and TILs entrapped within peritumoral fibrotic areas expressing *IDO1*, *PD-L1*, and *β-catenin* (*CTNNB1*) [65]. Qin et al. [60] confirmed the more immunosuppressive TME in M-UM and found intra-tumoral rather than peripheral CD8+ infiltrates. However, a study considering 35 archival formalin-fixed, paraffin-embedded M-UM specimens described a tumor microenvironment in which M2-macrophages were the dominant subtype, CD4+ TILs were perivascular, and CD8+ lymphocytes were mainly peritumoral [59], suggesting that immune cells cannot invade the tumor to attack tumor cells. Recently, Coupland and coworkers classified UM hepatic metastases in four different groups: ‘absent/cold’ metastases with no TILs or TAMs in the tumor or at the tumor-normal liver interface, ‘altered immunosuppressive’ with a low scattered pattern of inflammatory cell infiltrate, ‘altered excluded’ where infiltrates of TILs or TAMs were low at the tumor center but high at the margin, and ‘high/hot’ where high infiltration of TILs or TAMs was present throughout the metastatic tissue [63]. The authors concluded that the predominant cell types present in M-UM and responsible for the immunosuppressed environment were M2-type TAMs and exhausted CD8+ TILs. Moreover, the absence of PD-L1 expression on UM tumors may explain the failure of anti-PD-1 monotherapy [60,63]. Indeed, several reports [26,59,62,65] and our unpublished observations find an elevated infiltration of CD8+ TIM-3+ and LAG-3+, but PD-1 negative cells suggesting that immune resistance in UM may occur via alternative immune checkpoints.

MART-1 and/or gp100 antigen-specific T cells were expanded in vitro from biopsy-derived TILs with IL-2. T cells displayed exhausted phenotype (PD-1+, CD39+, TIM-3+, TIGIT+, and LAG-3+) [26]. Similar results were obtained using single-cell (sc)RNA-sequencing by Durante et al. [62], who detected clonally expanded T cells and/or plasma cells in UM samples. Altogether, these data indicate that TILs may have mounted a response, despite the low tumor mutational burden.

TILs from a subset of a total of 13 UM patients have been identified and showed robust anti-tumor reactivity, similar to that frequently observed in TILs from CM patients. Interestingly, the number of TILs recovered from UM and CM were similar, but after two weeks of culture in the presence of IL-2, UM-derived TIL cultures were mainly CD4+T cells and produced IFN-γ in response to parental tumor cells [66]. In another setting, TILs from UM metastases from 5 patients were successfully expanded in vitro applying an agonistic anti-4-1BB and OKT3 antibodies (anti-CD3) with high dose IL-2 in a small device to produce immune cells for clinical use. The authors report that this method allows the proliferation of TILs in a short time frame, and TILs obtained after such expansion were mostly CD8+, not overly differentiated. The ability of these TILs to recognize and respond to autologous tumor cells was successfully pursued by the authors only in one case where TILs produced a discrete amount of IFN-γ [67].

The efficacy of in vitro expanded autologous TILs from UM metastasis in patients was addressed in a phase II clinical trial (ClinicalTrials.gov Identifier: NCT01814046) enrolling a total of 20 patients. Reinfusion of TILs after a non-myeloablative lymphodepleting conditioning regimen could induce objective tumor regression in 7/20 (35%) M-UM patients. Among the responders, one highly pre-treated patient demonstrated a durable complete regression of numerous hepatic metastases for two years (Table 1) [68]. Johansson et al. [69] found a direct correlation between the high infiltration of CD8+ T cells and macrophages with longer overall survival in patients before treatment with hyperthermic isolated hepatic perfusion (IHP). This is the only report indicating a positive correlation between the presence of immune cells and survival, although this may be related to the low numbers of metastatic biopsies studied.

In summary, both P- and M-UM TILs display a phenotype mostly immunosuppressive or exhausted, and subsets of M-UM patients possess TILs that are antigen-specific and thus may potentially be responsive to immunotherapy. The use of antibodies/inhibitors of appropriate immune checkpoint expressed by M-UM may be the therapeutic option to be pursued, at least in a subset of UM patients.

### 2.4. Alternative Immune Checkpoint

The PD-1/PD-L1 immune checkpoint seems not to be as frequently upregulated in UM as in CM metastases; therefore, criticism on the strength of the rationale for this checkpoint blockade in UM has been raised [61]. Consistently, results from clinical trials with anti-PD-1 ICB are not so brilliant for M-UM patients [72]. This stimulated the search for new immune checkpoints, exhaustion markers, or immunosuppressive molecules that may become potential targets to be studied in clinical trials. The expression of the immunosuppressive molecule, IDO, and multiple immune checkpoint molecules, such as Vista, TIGIT, and LAG-3 on TILs in UM metastases has been shown [26,65]. TILs isolated from metastases and expanded in vitro, analyzed by flow cytometry, displayed in several cases tumor-reactive subsets of immune cells expressing the checkpoint receptors PD-1, TIM-3, LAG-3, and, to some extent, TIGIT [26]. The dominant exhaustion marker identified in UM was LAG-3 as analyzed by scRNA-seq and immunohistochemistry (IHC). This explains at least in part the failure of checkpoint blockade targeting CTLA-4 and PD-1. LAG-3 was found expressed mainly on CD8+ T cells but was also detected on some CD4+ T cells, FOXP3+ regulatory T cells, NK cells, and macrophages/monocytes [62]. Fusion protein and inhibitors of LAG-3 are in development or already tested in clinical trials either as a single agent or in association with anti-PD-L1, in different cancers, including UM (ClinicalTrials.gov Identifier: NCT02519322) (Table 2) [78].

## 3. Immune Checkpoint Inhibitors: Retrospective, Real-World Studies, and Clinical Trials

There is no consensus on the standard treatment of UM, and the correct management of this disease remains a matter of discussion. To determine progression-free and overall survival benchmarks, Khoia et al. reported in 2019 [70] a meta-analysis of 912 M-UM patients from 29 trials published from 2000 to 2016. Among the selected trials, five studies used immunotherapy and only three of them used anti-CTLA-4. Considering the whole population, the median progression-free survival (PFS) was 3.3 months, the median overall survival (OS) was 10.2 months, and the 1-year OS rate was 43%. Liver-directed therapies appeared in this study as the best treatments.

UM is genetically and biologically different from CM [22,33] and is barely immunogenic due to its low number of mutations [6]. Surprisingly, a phase II trial (ClinicalTrials.gov Identifier: NCT01814046) with 21 M-UM patients treated with lymph-depleting chemotherapy (cyclophosphamide followed by fludarabine) and a single intravenous infusion of autologous TILs with high-dose IL-2, showed exciting results (Table 1) [68]. In this study, 7 (35%) patients demonstrated tumor regression, with 6 (30%) achieving a partial response (PR) and 1 achieving complete response (CR) (5%), justifying further investigations of other immunological approaches. A subsequent clinical trial with autologous TILs and IL-2 therapy in M-UM (ClinicalTrials.gov Identifier: NCT03467516) and another one in metastatic CM and UM (ClinicalTrials.gov Identifier: NCT00338377) are ongoing (Table 2).

An interesting approach is the use of dendritic cell (DC) vaccination in an adjuvant setting. The immunologic responses after adjuvant DC vaccination were studied in an open-label phase II clinical trial with high-risk UM. An increase in OS was observed in patients with a tumor antigen-specific immune response [83]. In addition, a multicenter, randomized, two-armed, open-label phase III study is currently ongoing to evaluate the adjuvant vaccination with tumor RNA-loaded autologous DC in patients with resected monosomy 3 UM (ClinicalTrials.gov Identifier: NCT01983748) (Table 2**)**. A phase I trial is studying the side effects and best dose of a modified virus called Vesicular Stomatitis Virus, VSV-IFNbetaTYRP1 in patients with stage III-IV melanoma including M-UM (ClinicalTrials.gov Identifier: NCT03865212) (Table 2). The VSV has been modified to express two extra genes: *IFN-beta* and *TYRP1*. IFN-β may protect normal healthy cells from becoming infected with the virus and improve the antitumor efficacy due to its intrinsic antiproliferative effects and tyrosinase-related protein 1 (TYRP1) is a tumor-associated antigen expressed both in CM and UM.

Single ICB, anti-CTLA-4, or anti-PD-1 therapy gave only limited results in terms of efficacy in patients with M-UM with an overall response rate (ORR) that ranged from 0.5 to 6% [84]. Better results were expected from the combination of the two monoclonal antibodies. A real-world study [74] analyzed retrospectively 9 UM patients treated with low-dose anti-CTLA-4 (Ipilimumab, ipi) (1 mg/kg) and standard-dose anti-PD-1 (Pembrolizumab) (2 mg/kg). Median OS was 18.4 months with neither CR nor PR (0/9). No deaths for treatment-related adverse events occurred; however, 18% of patients had at least one grade 3 or 4 toxicity (Table 1).

A retrospective analysis by Klemen et al. [71] reported a single institutional experience using antibodies against CTLA-4, PD-1, and/or PD-L1 to treat 428 patients with metastatic melanoma histologically diagnosed as cutaneous, unknown, acral, mucosal, or uveal. For the 30 patients with M-UM, median OS was 12.2 months, and 5-year OS was 22%. Most of the longer survivors received both anti-CTLA-4 and anti-PD-1 or anti-PD-L1 either sequentially or in combination (Table 1). Clinical retrospective data of 126 patients diagnosed with M-UM in Denmark were analyzed before (pre-ICB, *n* = 32) and after (post-ICB, *n* = 94) the approval of first-line treatment with ICB [72]. The study shows a significant improvement of survival in patients post-ICB therapy: the combined ICB treatment (19 patients) achieved 18.9 months median OS and 57.6% of 1-year OS rate (Table 1). A multi-center retrospective study [73] analyzed 64 M-UM patients, 50 of which received combined checkpoint blockade as first-line systemic therapy. The median PFS was 3.0 months and the median OS was estimated to 16.1 months with an ORR of 15.6%. Severe treatment-related adverse events were experienced by 39.1% of patients (Table 1). 

These retrospective studies showed better results than those obtained with Ipilimumab or anti-PD-1 (Nivolumab) monotherapy and established the basis for prospective clinical trials. At present, March 2021, there are 7 clinical trials involving combination immunotherapy listed by www.clinicaltrials.gov (Table 3).

A phase I pilot study (ClinicalTrials.gov Identifier: NCT03922880) plans to combine arginine depletion and ICB. Four phase I/II trials combine local liver therapy or immunoembolization with systemic administration of Ipilimumab and Nivolumab (Table 3). An open-label phase I basket study (ClinicalTrials.gov Identifier: NCT00986661, Table 2) is evaluating the safety and preliminary efficacy of intra-lesion PV-10 in patients with solid tumors of the liver including UM metastases. PV-10, a small molecule that accumulates in lysosomes inducing autolysis, can produce immunogenic cell death and therefore a T cell-mediated immune response against immunologically cold tumors, providing a rationale for the association with ICBs. Preliminary results were presented for 13 patients with stable disease (SD) in 62.5% and PR in 37.5% of patients [85]. Results from combination therapy with PV-10 and ICBs are awaited with interest. Complete results of the Spanish GEM-1402 study (ClinicalTrials.gov Identifier: NCT02626962) were recently published [75] (Table 1 and Table 3). This phase II trial tested the efficacy of the combination of Nivolumab and Ipilimumab as first-line therapy in 52 patients with M-UM. Median OS was 12.7 months with a median PFS of 3 months. The outcome seems quite modest compared to benchmarks of UM responses. The authors claim that the short PFS may be related to the high levels of LDH, a serum marker of progression, at baseline. *GNAQ*, *GNA11*, and *SF3B1* gene mutational analysis and Multiplex Ligation Probe Amplification (MLPA) analysis to detect deletions and duplications in chromosomes 3 and 8 were performed in 25 patients (50% of total patients). Mutations and chromosomal aberrations did not appear to be related to ORR, although the number of patients analyzed was too small to obtain conclusive results. Treatment-related adverse events occurred in 49 of 52 patients with 1 death in a patient with thyroiditis and 1with Guillain–Barrè syndrome. Pelster et al. [76] (ClinicalTrials.gov Identifier: NCT01585194, PROSPER), reported on a phase II study of Nivolumab plus Ipilimumab an ORR of 18%, a median PFS of 5.5 months, and a median OS of 19.1 months in 33 patients, which is longer than the 6.8 to 9.6 months reported with monotherapy. Grade 3–4 treatment-related adverse events occurred in 40% of patients (Table 1 and Table 3).

The influence of *BAP1* mutation or chromosome 3 monosomy has been considered only in a small fraction of patients. Patients at high risk of metastasis with monosomy 3 and/or *BAP1* mutation should be included in clinical trials of adjuvant therapy. Considering only clinical trials (www.clinicaltrials.gov) with updates starting in 2019 to March 2021, we found 7 clinical trials using adjuvant therapy in high-risk UM patients. One uses ICBs, and 4 other immunological approaches, whereas 2 exploit targeted therapies (Table 2). Interestingly, a randomized phase II study enrolling resectable metastatic melanoma including UM, uses Ipilimumab, Nivolumab, and Relatlimab, the latter blocking LAG-3 (ClinicalTrials.gov Identifier: NCT02519322, Table 2).

In summary, an increase in ORR was observed in combined ICB treatment compared to monotherapy although not comparable with the improvement obtained in CM.

A new approach is based on the bispecific soluble molecule Tebentafusp. This fusion molecule binds with high affinity the GP100 peptide presented by HLA-A*02:01 on tumor cells and, with the anti-CD3 effector domain, induces a polyclonal activation of naive T cells. Tebentafusp activates T cells independently of their natural TCR specificity. The phase I/II trial of Tebentafusp in metastatic melanoma (ClinicalTrials.gov Identifier: NCT01211262, Table 1 and Table 2) enrolling previously treated cutaneous (*n* = 61) and M-UM patients (*n* = 18) recently reported a one-year OS rate of 65%, 16.6% PR, and 44.4% SD [77]. IFN-γ related markers (CXCL10, CXCL11, IL6, IL10, IL15, and IFN-γ) were measured in the serum at baseline and on treatment, and an increase was found in 11/18 UM patients analyzed. At present, March 2021, 2 additional clinical trials studying Tebentafusp are listed in www.clinicaltrials.gov (https://clinicaltrials.gov/ct2/results?term=Tebentafusp+and+uveal+melanoma&Search=Search): 1 phase II randomized, open-label, multicenter study in untreated, advanced UM (ClinicalTrials.gov Identifier: NCT03070392) and 1 phase I/II (ClinicalTrials.gov Identifier: NCT02570308) intra-patient escalation dosing in advanced UM (Table 2). Preliminary results were presented at the ESMO Immuno-Oncology Virtual Congress 2020. Following Tebentafusp, the ORR was 5% with only PRs. Stable disease was achieved by 45% of patients. The median duration of response was 8.7 months. With a median follow-up of 19.6 months, the median OS was 16.8 months. Patients (64%) developing rash within 7 days of Tebentafusp initiation demonstrated a superior median OS of 22.5 months compared to 10.3 months in patients with no rash, suggesting an immune-related effect. Further results need to clarify a real improvement in survival by bispecific molecules, although the U.S. Food and Drug Administration (FDA) has granted breakthrough therapy designation to Tebentafusp (IMCgp100 for HLA-A*02:01-positive patients) in UM [86].

The expression of Preferentially expressed Antigen in Melanoma (PRAME) correlates with high metastatic risk in UM [87] and is presently under investigation in several clinical trials as an immunotherapeutic target antigen of M-UM (ClinicalTrials.gov Identifier: NCT04262466 and NCT02743611, Table 2). In ClinicalTrials.gov Identifier: NCT04262466, a bispecific molecule consisting of a TCR targeting HLA-A*02:01 plus PRAME and anti-CD3 scFv will be used in association with anti-PD-L1 to treat PRAME positive patients. ClinicalTrials.gov Identifier: NCT02743611 exploits participants T cells that are modified to recognize and target PRAME on cancer cells.

The identification of an immunotherapy response signature would be of great advantage to spare potential non-responders from elevated toxicity. A recent attempt to identify molecular markers of immunotherapy resistance of metastatic CM was reported by Beck et al. using a clinical proteomic approach [88].

## 4. Immune Signatures

Several studies have characterized the immune infiltrate in metastatic UM, given the crucial prognostic role of TME in various types of metastatic cancers. Immune prognostic signatures have been proposed to identify those patients who could benefit from immunotherapy in an attempt to reduce the 5-year mortality rate. These signatures have been developed by digital cytometry working on retrospective, public datasets and require experimental validation to verify diagnostic reliability and clinical usefulness [89,90,91,92] (Table 4).

**Figure 1:**
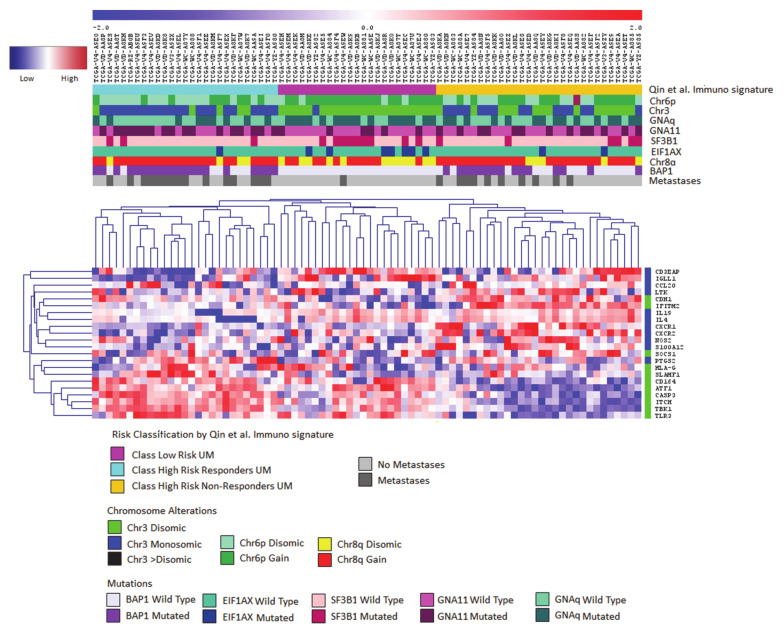
Application of the prognostic adaptive immune response signature developed by Qin et al. [60] to the TCGA-UM dataset. Euclidean hierarchical cluster Heatmap for 80 P-UM, highlighting mRNA expression levels of Qin et al. [60] immune signature genes. Responder genes are labeled in green, non-responder genes are labeled in blue. The expression values are reported by a color scale (blue = expression below the mean, red = expression above the mean, white = expression at the mean; the intensity is related to the distance from the mean). This signature shows three main clusters defined by differentially expressed profiles between responders versus non-responders genes.

Patel’s group recently proposed a study on a dataset of 47 P- and M-UM demonstrating, by IHC, that metastatic patients show significantly higher levels of immune infiltrate (CD3+, CD8+, FoxP3+, and CD68+ cells) compared to primary tumors [60]. They developed an IFN-γ signature using Nanostring technology between 2 responder and 4 non-responder patients to immunotherapy. Their data indicated that pre-treatment tumors of non-responders display a gene expression profile consistent with pro-inflammatory signaling, while responders have significantly elevated levels of *Suppressor Of Cytokine Signaling 1* (*SOCS1*) and *HLA* molecules. Two sets of genes that are differentially expressed between responders and non-responders were identified. Twelve genes were upregulated in responders at baseline before treatment and 13 showed significantly higher expression at baseline in non-responders. The authors identify, for the first time, a baseline tumor immune signature predicting response and resistance to immunotherapy in UM, that can be used to select those patients that are likely to respond to immunotherapy. A limit of this signature is the small number of patients (*n* = 6) analyzed. However, results from validation studies of this signature in larger cohorts of patients (GEM1402 and CA184-187) will provide more information on the resistance and response mechanisms of M-UM to immunotherapy, and prospective testing will establish clinical value. Figure 1 shows the application of this signature to the TCGA dataset of P-UM. The hierarchical clustering of this signature highlights three main clusters: high-risk with an immunotherapy responder profile (light blue), low-risk (pink), and high-risk non-responders (yellow). Among the high-risk UM, mostly metastatic cases with *BAP1* mutations, chromosome 3 monosomy, and chromosome 8q gain, 1 group (light blue) contains potential responders to immunotherapy.

## 5. Conclusions

Despite the considerable advancement in the diagnosis and classification of patients at low/high-risk of progression, UM still represents a challenge for oncologists. Indeed, still 50% of patients will develop metastatic disease with only marginal improvements in survival in decades. The origin from an immune-privileged site and the development of metastases in the liver, an immune-modulating organ, the low mutational burden, the few neoantigens, and the low expression of PD-L1 on tumor cells contribute to the poor response of UM to immunotherapy, compared to CM.

An increase in ORR was observed in patients receiving combined Ipilimumab and Nivolumab compared to monotherapy. The results achieved in UM are far from being comparable with the improvement obtained in CM, yet they are equal in terms of side effects. One of the reasons for this result is certainly the low expression of PD-1/PD-L1 in UM. Targeting LAG-3 that is expressed in UM at higher levels than PD-1 might yield better results. Immunotherapy is not only ICB treatment, and many different approaches are in development or already in clinical trials. Among these, Tebentafusp seems promising, since also the FDA has granted breakthrough therapy designation in UM. Yet, this treatment will be available for a small portion of patients because the drug is designed only for HLA-A*02:01-positive patients. This is a big issue, but it is strictly connected with immunotherapies that may require personalized drugs.

Single-cell omics studies and high-throughput data analysis are necessary and need to be improved to understand the mechanisms underlying the cross-talk between tumor and immune cells. This approach will provide new insights and identify new potentially actionable targets for immunotherapy. UM express few neo-antigens but high levels of TAA, such as MART1, GD2, Tyrosinase1, TRP1, gp100, and MAGE. Cell-based immunotherapies that are being developed exploit some of these TAA as targets. A phase I study using GD2-directed Chimeric Antigen Receptor T cells (CAR-T cells) is ongoing in patients of different cancers, including UM (ClinicalTrials.gov Identifier: NCT03635632, Table 2). Another antigen that has been successfully targeted by immunotherapy is PRAME either with bispecific TCR/anti-CD3 molecules (ClinicalTrials.gov Identifier: NCT04262466) or with autologous T cells engineered with PRAME-specific TCR (ClinicalTrials.gov Identifier: NCT02743611). Local liver chemotherapy and radiotherapy may release neoantigens and soluble mediators attracting cells from the immune system, into the tumor. The association of selective internal hepatic radiation (microspheres containing radioactive yttrium-90) with the combination of Ipilimumab and Nivolumab is exploited in an interventional open-label phase I/II clinical trial (ClinicalTrials.gov Identifier: NCT02913417, Table 2).

Recent observations highlight the expression of alternative immune-checkpoints: LAG-3 and TIM-3 should preferentially be targeted instead of PD-1/PD-L1, which are barely expressed by UM metastases. The clinical trial ClinicalTrials.gov Identifier: NCT02519322 that uses the association of anti-LAG-3 with Ipilimumab and Nivolumab and is, at present, enrolling patients will eventually show the advantage of LAG-3 targeting (Table 2).

Most studies exploiting new possibilities for ICB associations could be done in vitro in an autologous setting if lymphocytes and cells from the same patient were available. Syngeneic murine models are so far inappropriate, since many of them are obtained using melanoma cell lines to generate liver metastasis, thus resembling neither the biology nor the genetics of UM. Patient-Derived Xenografts (PDX), injected either subcutaneously or orthotopically, are also challenging to develop for M-UM, and they may be useful to test the tumor response to pharmacological or targeted therapy rather than to immunotherapy, since PDX cannot maintain immune cells alive. Humanized mice may be used to overcome this issue. Finally, the use of organoids, in vitro 3D culture systems, that keep the biological characteristics of the original tumor to simulate the in vivo tumor growth may be a useful method to study the effects of drugs before they come to the clinic.

## Figures and Tables

**Table 1:** Immunotherapy in UM: published studies.

Study	Type of Study	Targeted Patients	No Patients (UM Patients)	ORR	Median OS	Median PFS	Rate 1-Year Surv	6 Months PFS	PR	CR	SD
Khoia [70]	Meta-analysis (2000–2016)	metastatic uveal melanoma	(912)	-	10.2	3.3	43%	27%	-	-	-
Chandran [68]	Phase II ClinicalTrials.gov Identifier: NCT01814046 (autologous TILs)	Metastatic Ocular Melanoma Metastatic Uveal Melanoma	(21)	NE	NE	NE	NE	NE	30%	5%	NE
Klemen [71]	Retrospective(Ipilimumab+Nivolumab)	metastatic melanoma	428 (30)	-	12.2	-	-	-	-	-	-
Bol [72]	Retrospective (Ipilimumab+Nivolumab)	metastatic UM	(126) Ipilimumab/Nivolumab *n* = 19l	-	18.9	3.7	57.6%	3.7	21.1%	0	10.5%
Heppt [73]	Retrospective (Ipilimumab+Nivolumab)	metastatic or unresectable UM	(64) Ipi+nivo55	15.6	16.1	3	-	-	-	-	-
Kirchberg [74]	Real world (Ipilimumab+Nivolumab)	metastatic melanoma	33(9)	-	18.4	-	-	-	0	0	56%
Piulats [75]	Phase II ClinicalTrials.gov Identifier: NCT02626962 (Ipilimumab+Nivolumab)	Metastatic uvela melanoma	(52)	-	12.7	3	51.9%	-	9.6%	1.9%	-
Pelster [76]	Phase II ClinicalTrials.gov Identifier: NCT01585194 (Ipilimumab+Nivolumab)	Metastatic uveal melanoma	(35)	18%	19.1	5.5	56%	-	15%	3%	33%
Middleton [77]	Phase I/II ClinicalTrials.gov Identifier:NCT01211262 (Tebentafusp)	Advanced melanoma	84 (19)	-	-	-	65%	-	16.6%	0	44.4%

Abbreviations: NE: not evaluated; ORR: overall response rate; PFS: progression free survival; surv: survival; OS: overall survival; PR: partial response; CR: complete response; SD: stable disease.

**Table 2:** Ongoing clinical trials with adjuvant and not adjuvant therapies in UM.

ClinicalTrials.gov Identifier: NCT Number	Trial (Adjuvant)	Status	Phase	Targeted Patients	Actual Enrollment (Estimated Enrollment)	Principal Investigator	First Submitted Date	Last Update Posted Date
NCT02068586	A Randomized Phase II Study of Adjuvant Sunitinib or Valproic Acid in High-Risk Patients With Uveal Melanoma	Recruiting	Phase II	Ciliary Body and Choroid Melanoma Iris Melanoma Intraocular Melanoma	(150)	Takami Sato	19 February 2014	7 January 2021
NCT02223819	Phase II Trial of Adjuvant Crizotinib in High-Risk Uveal Melanoma Following Definitive Therapy	Active, not recruiting	Phase II	Uveal Melanoma	34 (30)	Richard Carvajal	20 August 2014	18 December 2019
NCT01983748	A non-commercial, multicenter, randomized, two-armed, open-label phase III study to evaluate the adjuvant vaccination with tumor RNA-loaded autologous dendritic cells versus observation of patients with resected monosomy 3 uveal melanoma	Recruiting	Phase III	Uveal Melanoma	(200)	Beatrice Schuler-Thurner	17 September 2013	6 January 2020
NCT01100528 [79]	Adjuvant Therapy for Patients With Primary Uveal Melanoma With Genetic Imbalance (Dacarbazine+IFNa-2B)	Completed	Phase II	Iris, Ciliary Body or Choroidal Melanoma	38(36)	Yogen Saunthararajah	7 April 2010	26 February 2019
NCT02519322 [78]	Neoadjuvant and Adjuvant Checkpoint Blockade (Ipi+Nivo+Relatlimab)	Recruiting	Phase II	Cutaneous Melanoma Mucosal Melanoma Ocular Melanoma	(53)	Rodabe N Amaria	4 August 2015	30 December 2020
NCT00254397	Study of the Modulatory Activity of an LHRH-Agonist (Leuprolide) on Melanoma Peptide Vaccines as Adjuvant Therapy in Melanoma Patients	Completed	Phase II	Melanoma	98	Patrick Hwu	14 November 2005	16 October 2019
NCT01989572 [80]	A Randomized, Placebo-Controlled Phase III Trial of Yeast Derived GM-CSF Versus Peptide Vaccination Versus GM-CSF Plus Peptide Vaccination Versus Placebo in Patients With “No Evidence of Disease” After Complete Surgical Resection of “Locally Advanced” and/or Stage IV Melanoma	Completed	Phase III	Ocular melanoma Cutaneous Melanoma Mucosal melanoma	815	David H Lawson	18 November 2013	7 July 2020
**ClinicalTrials.gov Identifier:** **NCT number**	**Trial (other not adjuvant immunological therapies)**	**Status**	**Phase**	**Targeted Patients**	**Actual enrollment** **(Estimated enrollment)**	**Principal Investigator**	**First Submitted Date**	**Last Update Posted Date**
NCT03070392	A Phase II Randomized, Open-label, Multi-center Study of the Safety and Efficacy of IMCgp100 Compared With Investigator Choice in HLA-A*0201 Positive Patients With Previously Untreated Advanced Uveal Melanoma	Active, not recruiting	Phase II	Uveal Melanoma	378 (327)	Mohammed Dar	14 February 2017	6 January 2021
NCT02570308	A Study of the Intra-Patient Escalation Dosing Regimen With IMCgp100 in Patients With Advanced Uveal Melanoma	Active, not recruiting	Phase IPhase II	Uveal Melanoma	(150)	Not Provided	6 October 2015	6 January 2021
NCT03467516	A Phase II Study to Evaluate the Efficacy and Safety of Adoptive Transfer of Autologous Tumor-Infiltrating Lymphocytes in Patients With Metastatic Uveal Melanoma	Recruiting	Phase II	Uveal Neoplasms Melanoma, Uveal	(59)	Udai S Kammula	9 March 2018	18February 2020
NCT00986661	A Phase I Study to Assess the Safety, Tolerability, and Pharmacokinetics of PV-10 Chemoablation of Cancer Metastatic to the Liver or Hepatocellular Carcinoma Not Amenable to Resection or Transplant	Recruiting	Phase I	Cancer Metastatic to the Liver Hepatocellular Carcinoma Metastatic Melanoma Metastatic Ocular Melanoma Metastatic Uveal Melanoma Metastatic Lung Cancer Metastatic Colon Cancer Metastatic Colorectal Cancer Metastatic Breast Cancer Metastatic Pancreatic Cancer	(78)	Eric Wachter	24 September 2009	5 March 2020
NCT01211262 [77]	A Phase I, Open-Label, Dose-Finding Study to Assess the Safety and Tolerability of IMCgp100, a Monoclonal T Cell Receptor Anti-CD3 scFv Fusion Protein in Patients With Advanced Malignant Melanoma	Completed	Phase I	Malignant Melanoma	84(50)	Namir Hassan	28 September 2010	8 July 2020
NCT04262466	Phase I/II Study of IMC-F106C in Advance PRAME-Positive Cancers	Recruiting	Phase IPhase II	Select Advanced Solid Tumors	(170)	Shaad Abdullah, FACP	30 January 2020	16 February 2021
NCT02743611	A Phase I/II Dose-Finding Study to Evaluate the Safety, Feasibility, and Activity of BPX-701, a Controllable PRAME T-Cell Receptor Therapy, in HLA-A2+ Subjects With AML, Previously Treated MDS, or Metastatic Uveal Melanoma	Active, not recruiting	Phase IPhase II	Acute Myeloid Leukemia Myelodysplastic Syndrome Uveal Melanoma	28 (36)	Bellicum Pharmaceuticals Senior Director	11 April 2016	27 April 2020
NCT02697630 [81]	A Multicenter Phase II Open-Label Study to Evaluate Efficacy of Concomitant Use of Pembrolizumab and Entinostat in Adult Patients With Metastatic Uveal Melanoma	Active, not recruiting	Phase II	Metastatic Uveal Melanoma	(29)	Not Provided	22 February 2016	16 October 2019
NCT00338377 [82]	Lymphodepletion Plus Adoptive Cell Transfer With or Without Dendritic Cell Immunization in Patients With Metastatic Melanoma	Recruiting	Phase II	Melanoma	(189) 5 MU(primary site choroid)	Rodabe N. Amaria	10 February 2006	9 December 2020
NCT03635632	Phase I Study of Autologous T Lymphocytes Expressing GD2-specific Chimeric Antigen and Constitutively Active IL-7 Receptors for the Treatment of Patients With Relapsed or Refractory Neuroblastoma and Other GD2 Positive Solid Cancers(GAIL-N)	Recruiting	Phase I	Relapsed Neuroblastoma Refractory Neuroblastoma Relapsed Osteosarcoma Relapsed Ewing Sarcoma Relapsed Rhabdomyosarcoma Uveal Melanoma Phyllodes Breast Tumor	(94)	Bilal Omer	13 August 2018	9 December 2020
NCT03865212	Phase I Trial to Evaluate the Safety and Efficacy of Intratumoral and Intravenous Injection of Vesicular Stomatitis Virus Expressing Human Interferon Beta and Tyrosinase Related Protein 1 (VSV-IFNb-TYRP1) in Patients With Metastatic Ocular Melanoma and Previously Treated Patients With Unresectable Stage III/IV Cutaneous Melanoma	Recruiting	Phase I	Clinical Stage III Cutaneous Melanoma AJCC v8 Clinical Stage IV Cutaneous Melanoma AJCC v8 Metastatic Choroid Melanoma Metastatic Melanoma Metastatic Mucosal Melanoma Metastatic Uveal Melanoma Pathologic Stage III Cutaneous Melanoma AJCC v8 Pathologic Stage IIIA Cutaneous Melanoma AJCC v8 Pathologic Stage IIIB Cutaneous Melanoma AJCC v8 Pathologic Stage IIIC Cutaneous Melanoma AJCC v8 Pathologic Stage IIID Cutaneous Melanoma AJCC v8 Pathologic Stage IV Cutaneous Melanoma AJCC v8 Unresectable Melanoma	(72)	Roxana S Dronca	6 March 2019	18 November 2020

**Table 3:** Combination immunotherapies in UM.

ClinicalTrials.gov Identifier: NCT Number	Trial	Status	Phase	Targeted Patients	Actual Enrollment (Estimated Enrollment)	Principal Investigator	First Submitted Date	Last Update Posted Date
NCT01585194 [76]	Phase II Study of Nivolumab in Combination With Ipilimumab for Uveal Melanoma	Active, not recruiting	Phase II	Metastatic Uveal Melanoma Stage IV Uveal Melanoma AJCC v7	67 (141)	Sapna Patel	23 April 2012	10 December 2020
NCT02626962 [75]	Phase II Multicenter, Non-Randomized, Open-Label Trial of Nivolumab in Combination With Ipilimumab in Subjects With Previously Untreated Metastatic Uveal Melanoma	Active, not recruiting	Phase II	Uveal Melanoma	48 (48)	Josep Maria Piulats	1 December 2015	19 October 2020
NCT03922880	Pilot Study Combining Arginine Depletion and Checkpoint Inhibition in Uveal Melanomas	Active, not recruiting	Phase I	Uveal Melanoma	9 (9)	Alexander Shoushtari	18 April 2019	11 January 2021
NCT02913417	A Feasibility Study of Sequential Hepatic Internal Radiation and Systemic Ipilimumab and Nivolumab in Patients With Uveal Melanoma Metastatic to Liver	Recruiting	Phase IPhase II	Uveal Melanoma Hepatic Metastases	(26)	David R. Minor	21 September, 2016	25 August 2020
NCT04463368	SCANDIUM II Trial—A Phase I Randomized Controlled Multicentre Trial of Isolated Hepatic Perfusion in Combination With Ipilimumab and Nivolumab in Patients With Uveal Melanoma Metastases	Not yet recruiting	Phase I	Uveal Melanoma Liver Metastases	(18)	Roger Olofsson Bagge	5 July 2020	1 September 2020
NCT04283890	Phase Ib/2 Study Combining Hepatic Percutaneous Perfusion With Ipilimumab Plus Nivolumab in Advanced Uveal Melanoma	Recruiting	Phase IPhase II	Uveal Melanoma, Metastatic	(88)	Ellen W. Kapiteijn	21 February 2020	25 February 2020
NCT03472586	Ipilimumab and Nivolumab in Combination With Immunoembolization for the Treatment of Metastatic Uveal Melanoma	Recruiting	Phase II	Metastatic Uveal Melanoma	(35)	Marlana Orloff	14 March 2018	28 May 2020

**Table 4:** Summary of the principal published immune signature.

Ref.	Signature	Aim of the Study
Li [91]	Immune-related gene signature based on two immune-related genes for predicting survival in UM.	Development of an immune-related prognostic and predictive signature to identify those patients who could benefit from immunotherapy. The signature is built on the TCGA-UM dataset and is significantly associated with tumor T stage and tumor basal diameter.
Wang [90]	Adaptive Immune Resistance Signature based on fifteen markers, to predict prognosis in UM.	Analysis of the immune and stromal infiltrate on gene expression data of the TCGA-UM and TCGA-CM datasets using different digital cytometry algorithms for significant prognostic marker selection. This signature could identify UM subgroups with a characteristic tumor microenvironment.
Zhang [89]	Immune cell-based prognosis signature to predict overall survival in UM. The signature is based on the contribution of CD8+, CD4+ T cells, monocytes, and Mast cells.	Tumor microenvironment landscape analysis by the CYBERSORT algorithm to classify the immune cell type profiles in the TCGA-UM patients. This signature highlights the impact of immune infiltrate components in the development of metastases.
Gong [92]	Immune and stromal prognostic signature based on published datasets. The signature is developed on a four-cell model (cytotoxic, Th1, Th2 cells, and myocytes).	Tumor microenvironment analysis by ESTIMATE algorithm for the identification of a four-cell model as a biomarker of overall survival in UM. This prognostic signature can stratify subgroups of patients with different classes of risk.
